# PAR Interception and Utilization in Different Maize and Soybean Intercropping Patterns

**DOI:** 10.1371/journal.pone.0169218

**Published:** 2017-01-05

**Authors:** Xin Liu, Tanzeelur Rahman, Feng Yang, Chun Song, Taiwen Yong, Jiang Liu, Cuiying Zhang, Wenyu Yang

**Affiliations:** 1 College of Agronomy, Sichuan Agricultural University, Chengdu, China; 2 Key Laboratory of Crop Ecophysiology and Farming System in the Southwest, Ministry of Agriculture, Chengdu, China; 3 Sichuan Engineering Research Center for Crop Strip Intercropping System, Chengdu, China; 4 Institute of Ecological and Environmental Sciences, Sichuan Agricultural University, Chengdu, China; 5 Meteorological Bureau, Heze, China; Chinese University of Hong Kong, HONG KONG

## Abstract

The crop intercepted photosynthetically active radiation (PAR) and radiation use efficiency (RUE) vary markedly in different intercropping systems. The HHLA (horizontally homogeneous leaf area) and ERCRT (extended row crop radiation transmission) models have been established to calculate the intercepted PAR for intercrops. However, there is still a lack of study on the intercepted PAR and RUE under different intercropping configurations using different models. To evaluate the intercepted PAR and RUE in maize and soybean under different intercropping systems, we tested different strip intercropping configurations (SI1, SI2, and SI3 based on ERCRT model) and a row intercropping configurations (RI based on HHLA model) in comparison to monoculture. Our results showed that the intercepted PAR and RUE of intercropping systems were all higher than those of monoculture. The soybean intercepted PAR in strip intercropping was 1.35 times greater than that in row intercropping. In row intercropping (RI), the lack of soybean intercepted PAR resulted in a significant reduction of soybean dry matter. Therefore, it is not the recommended configuration for soybean. In strip intercropping patterns, with the distance between maize strip increased by 0.2 m, the intercepted PAR of soybean increased by 20%. The SI2 (maize row spacing at 0.4 m and the distance between maize strip at 1.6 m) was the recommended configuration to achieve the highest value of intercepted PAR and RUE among tested strip intercropping configurations. The method of dry matter estimation using intercepted PAR and RUE is useful in simulated experiments. The simulated value was verified in comparison with experimental data, which confirmed the credibility of the simulation model. Moreover, it also provides help in the development of functional-structural plant model (FSPM).

## Introduction

Intercropping is important for ample food supply in developing countries [1–2]. In the intercropping system, a tall statured crop is intercropped with a short statured crop, such as the cereal and legume intercropping [3–4]. The low yield of short statured intercrop, which is mainly contributed by the decrease of intercepted PAR, is often regarded as the limiting factor for the application of intercropping [4–5]. Due to high radiation use efficiency (RUE) and land equivalent ratio (LER), the strip intercropping is becoming more and more popular in different regions of the world [6–7].

The crop intercepted PAR varies greatly in different intercropping configurations, and positively correlate with the crop dry matter [8–11]. In maize and soybean intercropping, there are severe interspecific light competition between soybean and maize [12–13]. The soybean plants suffer shading effect of maize plants. Moreover, the maize plants miss PAR in the distance between adjacent maize strips [13]. The large distance between adjacent maize strips increases the intercepted PAR of understory soybean [6, 11].

There were two models previously used to estimate the intercepted PAR of intercrops. The HHLA (horizontally homogeneous leaf area) model, which divided the hybrid canopy into three parts [12–13]: the upper part of maize leaf (upper layer), the lower part of maize leaf (lower layer), and soybean leaf. The interception of these three parts can be divided by two steps. First is to distinguish the upper and lower layer, and then to differentiate the two lower layers. It was successfully used in the research of crop-weed competition [14] and intercropping systems [13, 15]. However, this model showed marked errors when applying to strip intercropping systems [6, 10, 11]. The RCRT (row crop radiation transmission) model proposed view factor method to calculate the interception in strip intercropping [10–11]. The strip-path factor [6, 11], which distinguished the incoming radiation transmitting to crop strip and blank path using integral methods, was considered in this model. However, there was no clear method to calculate the interception of lower part canopies. To address this issue, the ERCRT (extended RCRT) model was developed [6]. It divided the PAR into 9 layers of PAR intercepted fraction, and the divided PAR can be used to calculate the intercepted PAR of each intercrop species [6]. The models for intercepted PAR estimation in intercropping are gradually maturing. However, there was no study using these models to evaluate intercropping configurations with different row arrangements.

The RUE is quite different for crop species in different cropping systems. The RUE of tall statured intercrop is no more than that of its sole crop [11, 13, 16, 17]. However, the RUE of short statured intercrop is higher than that of its sole crop due to the diffused light effect and less light saturation [11, 13, 16]. The RUE of intercrops exhibits little variations under different crop canopy geometry according to the research of maize and soybean intercropping [13] with tall and short statured intercrop ratio of 1:3 and 2:3, and relay intercropping of wheat and its subordinate cotton [11] with row ratio of 3:1, 3:2, 4:2, and 6:2. There is still lack of knowledge on how the RUE changes in different intercropping configurations.

In LINTUL (light interception and utilization) model from Netherlands [18], the intercepted PAR and RUE can be used to estimate the dry matter accumulation. Others comprehensive models can also provide the accurate prediction of dry matter production, such as DSSAT (decision support system for agrotechnology transfer) from America [19–20], APSIM (agricultural production systems simulator) from Australia [21]. However, these models had no strip intercropping modules. The study on simulation of dry matter in different intercropping patterns can provide supports for the development of the strip intercropping modules.

A comprehensive research is needed to describe the intercepted PAR and RUE in different intercropping patterns, such as intercropping system with horizontally homogeneous leaf area and strip intercropping with different strip width. The objectives of this study were: (i) to evaluate intercepted PAR and RUE of intercropping crops with different patterns, including strips of different widths; (ii) to compare HHLA and ERCRT models in strip intercropping and homogeneous canopy intercropping configurations; (iii) to propose an alternative model to estimate crop dry matter in intercropping.

## Materials and Methods

### Ethics statement

No specific permits were required for the described field studies. All experiments were performed according to institutional guidelines of Sichuan Agricultural University, China.

### Site description

**Fig 1:**
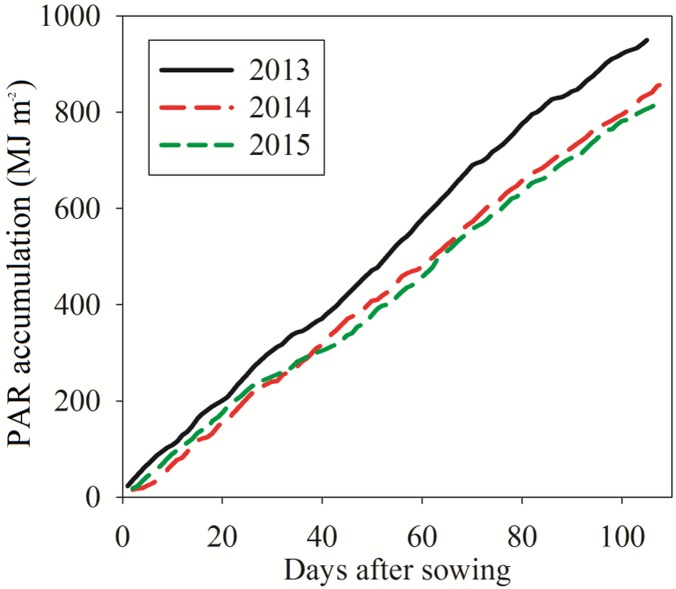
Incoming PAR accumulation during 2013, 2014 and 2015 growing seasons, in Heze city. Data were recorded from the local Meteorological Bureau.

Field experiments were conducted in Heze city (115°25′05″E, 35°15′09″N), Shandong province of China during 2013–2015. The climate of the region is temperate continental monsoon. The annual mean air temperature was 14.7°C with a frost-free period of 210 days. The meteorological data were collected from the local Meteorological Bureau in Heze city. Three years of accumulative radiation data (2013 to 2015) are shown in Fig 1. The surface soil was clayey with pH of 7.6, and the available N, P, and K content were 97, 33, and 190 mg kg^-1^ before sowing, respectively. The experiments was fully irrigated. Chemical control of weed and pest were followed by the local farmers.

### Experimental design

**Fig 2:**
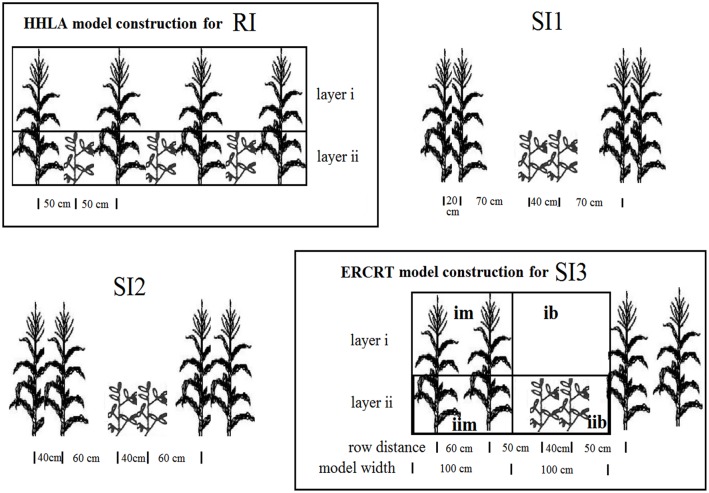
Row arrangement for treatments RI, SI1, SI2, and SI3. HHLA model construction for row intercropping (RI). ERCRT model construction for strip intercropping (SI), *e*.*g*. SI3.

The field experiments had six treatments arranged in randomized complete block with three replicates. The treatments include (Fig 2): (1) sole maize (SM): the row distance was 0.7 m, plants distance in rows was 0.2 m; (2) sole soybean (SS): the row distance was 0.5 m, plants distance in rows was 0.14 m; (3) row intercropping (RI): 1 row of maize intercropped with 1 row of soybean; (4) strip intercropping (SI1, SI2 and SI3): 2 rows of maize intercropped with 2 rows of soybean with different row arrangements (Fig 2). In SI1, strip intercropping with maize model width (*W*_*m*_): soybean model width (*W*_*s*_) was 60:140 (maize row apart to 20 cm). In SI2, strip intercropping with *W*_*m*_: *W*_*s*_ was 80:120 (maize row apart to 40 cm). In SI3, strip intercropping with *W*_*m*_: *W*_*s*_ was 100:100 (maize row apart to 60 cm). The size of each experimental plot was 6 m × 6 m, which had three continuous bands of SI and six continuous bands of RI. The crop densities were 7.1 plant m^-2^ for both monocultured and intercropped maize, and 14.2 plant m^-2^ for both monocultured and intercropped soybean. East-west row orientation was used in this study. The soybean and maize were sown on June 8–12^th^ and harvested on September 26–29^th^ for both intercropping and monoculture in three experimental years. Maize cultivar was “Xundan 26”, which was a compact type summer maize with 245 cm crop height. Soybean cultivar was “Hedou 19”, which was a local high yielding type with 67 cm plant height.

### Measurements

The leaf area index (*LAI*), crop height, and above ground dry matter of maize and soybean were measured every 7–13 days in each year. In model construction, the maize leaves were assumed to extend 20 cm on two sides. Ten soybean plants and five maize plants were sampled at each collection point. Samples from the intercropping boundary were avoided. The crop height of maize and soybean was measured without straightening them. To estimate *LAI*, single leaf area of soybean and maize were determined by leaf length×greatest leaf width×crop-specific coefficient (0.75 for soybean and 0.70 for maize). Before weighing, all samples of maize and soybean plants were dried to constant mass at 80°C in a drying oven.

To calculate extinction coefficient of maize (*k*_*m*_) and soybean (*k*_*s*_), the PAR were measured three times per plot in SM and SS on the same day of *LAI* measurement. The data were taken at canopy level and soil level at 9:00 am, 12:00 pm, and 15:00 pm [22]. The PAR intercepted fraction (*F*) was averaged from three measurements. *k* of maize and soybean were calculated as follows [13]:

$$k=-\frac{ln(1-F)}{LAI}$$ (1)

The sensor was positioned at three measuring points in parallel to row direction at the top of crop canopy and 5 cm above the soil. The *k*_*m*_ and *k*_*s*_ were 0.42 and 0.75 based on measured data (S1 Table). The measurements were obtained using a LI-191SA quantum sensor (LI COR Inc., 114 Lincoln, NE, USA) and a LI-1400 data logger. Incoming radiation values were provided by the Meteorological Bureau for the 2013 to 2015 growing seasons (Fig 1).

### Model description

#### Calculation of intercepted PAR

The model construction for both HHLA model and ERCRT model are shown in Fig 2, *e*.*g*. RI and SI3.

#### HHLA model (horizontally homogeneous leaf area)

The leaf distribution was horizontally homogeneous in the row intercropping configuration in our study. For calculate the proportion of intercepted PAR for the intercrops in RI [12–13], firstly, the following equation was used to separate the PAR intercepted faction of the layer *i*, which was the upper part of maize (*F*_*m-upper*_):

$$F_{m-upper}=1-\text{exp}(-k_{m}L_{m-upper}\text{)}$$ (2)

Secondly, the equations were used to divided the PAR intercepted faction for lower maize canopy (*F*_*m-lower*_) and soybean canopy (*F*_*s*_) in layer *ii*:

$$F_{s}=\frac{k_{s}L_{s}}{k_{m}L_{m-lower}+k_{s}L_{s}}\times \;[1-\text{exp}(-k_{m}L_{m-\text{lower}}-k_{s}L_{s})]$$ (3)

$$F_{m-lower}=\frac{k_{m}L_{m-lower}}{k_{m}L_{m-lower}+k_{s}L_{s}}[1-\text{exp}(-k_{m}L_{m-lower}-k_{s}L_{\text{s}})]$$ (4)

Where *L*_*s*_ and *L*_*m-lower*_ were the *LAI* for soybean and lower layer of maize, respectively. *L*_*m-upper*_ and *L*_*m-lower*_ were calculated by:

$$L_{m-upper}=\frac{h_{m}-h_{s}}{h_{m}}\times L_{m}$$ (5)

$$L_{m-lower}=\frac{h_{s}}{h_{m}}\times L_{m}$$ (6)

Where *h*_*m*_ was the maize height, and *h*_*s*_ was the soybean height. *L*_*m*_ was the total maize *LAI*.

#### ERCRT model (extended row crop radiation transmission)

There was large distance between adjacent maize strips in the strip intercropping systems. The ERCRT model (extended row crop radiation transmission) was used to evaluate the crop intercepted PAR in different strip intercropping configurations. *im* was the first layer of maize, and *ib* was the space above soybean canopy. *iim* and *iib* were the lower layer of maize and all soybean layer, respectively. The model was based on view factor theory [6, 10, 11], which was used to calculate crop interception in strip intercropping configuration.

**Fig 3:**
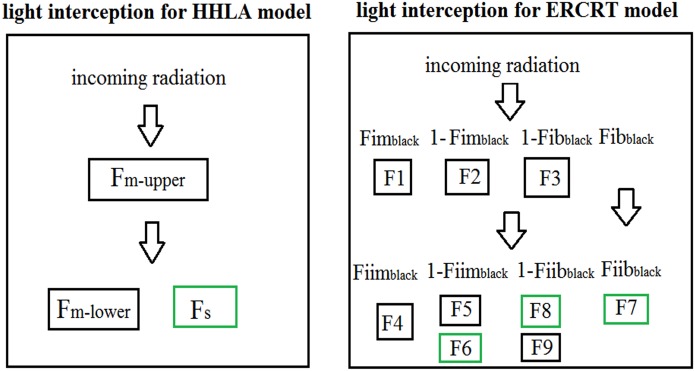
Fraction of intercepted PAR in HHLA and ERCRT models. The black frame indicated intercepted PAR by maize. The green frame indicated for intercepted PAR by soybean.

Following Wang *et al*. [6], we chose the equations which divided the intercepted PAR faction into nine parts (Fig 3):

$$F_{1}=f_{m}{Fim}_{black}[1-\text{exp}(-k_{m}L_{m-upper}/f_{m})]$$ (7)

$$F_{2}=f_{m}\{(1-{Fim}_{black})\;[1-\text{exp}(-k_{m}L_{m-upper})]\}$$ (8)

$$F_{3}=f_{s}\{(1-{Fib}_{black})[1-\text{exp}(-k_{m}L_{m-upper})]\}$$ (9)

$$F_{4}=Fim{Fiim}_{black}[1-\text{exp}(-k_{m}L_{m-lower})/f_{m}]$$ (10)

$$F_{5}=Fim\;\{(1-{Fim}_{black})\;[1-\text{exp}(-k_{m}L_{m-lower}-k_{s}L_{s})]\frac{k_{m}L_{m-lower}}{k_{m}L_{m-lower}+k_{s}L_{s}}\}$$ (11)

$$F_{6}=Fim\left\{\left(1-Fim_{black}\right)\;\left[1-\text{exp}\left(-k_{m}L_{m-lower}-k_{s}L_{s}\right)\right]\frac{k_{s}L_{s}}{k_{m}L_{m-lower}+k_{s}L_{s}}\right\}$$ (12)

$$F_{7}=Fib{Fiib}_{black}[1-\text{exp}(-k_{s}L_{s}/f_{s})]$$ (13)

$$F_{8}=Fib\;\{(1-{Fib}_{black})\;[1-\text{exp}(-k_{m}L_{m-lower}-k_{s}L_{s})]\frac{k_{s}L_{s}}{k_{m}L_{m-lower}+k_{s}L_{s}}\}$$ (14)

$$F_{9}=Fib\;\{(1-{Fib}_{black})\;[1-\text{exp}(-k_{m}L_{m-lower}-k_{s}L_{s})]\frac{k_{m}L_{m-lower}}{k_{m}L_{m-lower}+k_{s}L_{s}}\}$$ (15)

Where *f*_*m*_ was the area proportion of maize strip and soybean strip, and *f*_*s*_ was that of soybean strip, *e*.*g*. *f*_*m*_ and *f*_*s*_ were 0.5 and 0.5 in SI3, respectively. *Fim*_*balck*_ and *Fib*_*balck*_ were the view factors for first layer under of maize and soybean shape model, respectively. *Fiim*_*balck*_ and *Fiib*_*balck*_ were the view factors for second layer under of maize and soybean shape model, respectively (S1 Fig). The view factor was previously proposed to be calculated by the spatial integrating of any point on a lower plane [23]. The *Fim*_*black*_, *Fib*_*black*_, *Fiim*_*black*_ and *Fiib*_*black*_ were calculated by [6]:

$${Fim}_{balck}=\frac{\sqrt{{\left(h_{m}-h_{s}\right)}^{2}+W_{\text{m}}{}^{2}}-\;(h_{m}-h_{s})}{W_{\text{m}}}$$ (16)

$${Fib}_{balck}=\frac{\sqrt{{\left(h_{m}-h_{s}\right)}^{2}+W_{\text{s}}{}^{2}}-(h_{m}-h_{s})}{W_{\text{s}}}$$ (17)

$${Fiim}_{balck}=\frac{\sqrt{h_{s}{}^{2}+W_{\text{m}}{}^{2}}-h_{s}}{W_{\text{m}}}$$ (18)

$${Fiib}_{balck}=\frac{\sqrt{h_{s}{}^{2}+W_{\text{s}}{}^{2}}-h_{s}}{W_{\text{s}}}$$ (19)

*W*_*m*_ was the strip width of maize, and *W*_*s*_ was that of soybean, *e*.*g*. *W*_*m*_ and *W*_*s*_ were 1 m and 1 m in SI3, respectively. The PAR faction at the bottom of *im* and *ib* (S2 Fig) can be calculated by [6]:

$$Fim=f_{m}\;[\;{Fim}_{blacck}\times \text{exp}(-k_{m}L_{m-upper}/f_{m})]+f_{m}\;(1-{Fim}_{black})\times \text{exp}(-k_{m}L_{m-upper})]+f_{m}f_{s}\;(1-{Fib}_{black})\times \text{exp}(-k_{m}L_{m-upper})$$ (20)

$$Fib=f_{s}\;[{Fib}_{black}+f_{s}\;(1-{Fib}_{black})\text{ exp}(-k_{m}L_{m-uppe\text{r}})]+f_{m}f_{s}\;[(1-{Fim}_{black})\times \text{exp}(-k_{m}L_{m-upper})]$$ (21)

The PAR intercepted fractions of maize (*F*_*m*_, S3 Fig) and soybean (*F*_*s*_, S4 Fig) can be calculated by:

$$F_{m}=F1+F2+F3+F4+F5+F9$$ (22)

$$F_{s}=F6+F7+F8$$ (23)

#### Calculation of RUE

Radiation use efficiency (RUE) was established as follows [13, 15]:

$$RUE=\frac{ADM}{\Sigma I_{0}F}$$ (24)

*ADM* was the accumulated dry matter of intercrops (g m^-2^). The *I*_*0*_ was the quantity of daily incident PAR (MJ m^-2^). In order to convert total radiation to PAR, the daily total radiation data were multiplied by 0.5 [11, 22]. The *F* represented the fraction of intercepted PAR of the intercropped maize or soybean on certain days, which can be relevant to *I*_*0*_ and used to calculate the cumulative intercepted PAR of intercrop. The radiation use efficiency in this study means the photosynthetic active radiation (PAR) use efficiency.

#### Simulation of dry matter

The RUEs of intercropped maize and soybean were calculated in 2013 and 2014. Then, the *ADM* was simulated and validated in 2015.

$$ADM=I_{0}\;F\;RUE$$ (25)

#### Model validation

The root mean square error (*RMSE*) and mean bias error (*MBE*) were used to validate the models [15].

$$RMSE=\sqrt{\frac{1}{n}\sum_{i=1}^{n}{\left(y_{i}-x_{i}\right)}^{2}}$$ (26)

$$MBE=\frac{1}{n}\sum_{i=1}^{n}\left(y_{i}-x_{i}\right)$$ (27)

*x*_*i*_ and *y*_i_ are the measured and calculated values and *n* is the number of paired set data. The perfect model fit has *MBE* = *RMSE* = 0.

#### Statistical analysis

The statistical analyses were performed using SPSS software (version 19.0, SPSS Inc., Chicago, USA). All experiments were performed at least three times independently. Data are presented as mean ± standard deviation (SD). Statistical significance was determined using one way ANOVA. Duncan’s multiple range test (DMRT) was applied to compare the significant differences between treatments. A value of *P* < 0.05 was considered statistically significant.

## Results

### Dynamics of leaf area index and crop height

**Fig 4:**
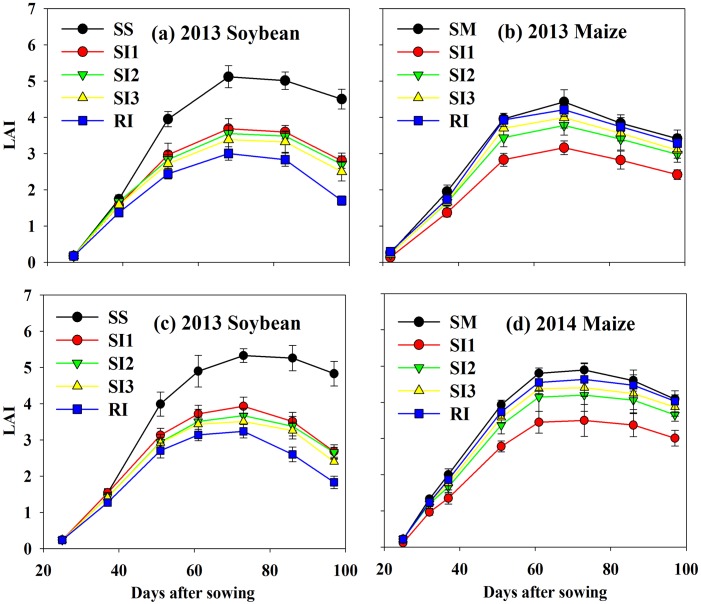
*LAI* dynamics of intercropped maize (b, d) and soybean (a, c) during the growing seasons of 2013 and 2014. RI: one row maize intercropped with one row soybean; SI1: strip intercropping with maize model width (*W*_*m*_): soybean model width (*W*_*s*_) of 60:140 (maize row of 20 cm); SI2: strip intercropping with *W*_*m*_: *W*_*s*_ of 80:120 (maize row of 40 cm); SI3: strip intercropping with *W*_*m*_: *W*_*s*_ of 100:100 (maize row of 60 cm); SS: sole soybean; SM: sole maize. Bars at each data point represent standard deviation of the mean (n = 3).

The *LAI* of maize and soybean system increased rapidly from 40–60 DAS (days after sowing), and reached to peak around 70 DAS. Across years and treatments, the maximum *LAI* of soybean in monoculture (SS) was 1.37, 1.45, 1.51, and 1.68 times higher than that of SI1, SI2, SI3, and RI, respectively (Fig 4a and 4c). For maize, the maximum *LAI* in monoculture (SM) was 1.20, 1.11, 1.08, and 1.05 times higher than that of SI1, SI2, SI3, and RI, respectively (Fig 4b and 4d).

**Fig 5:**
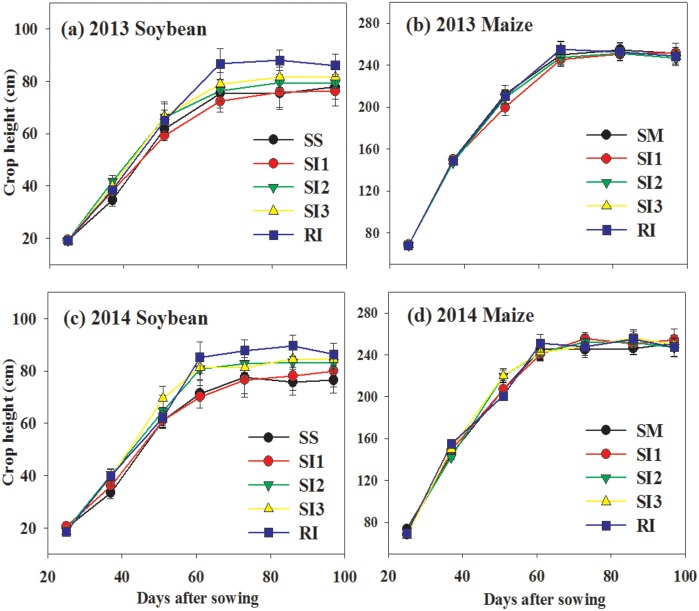
Crop height of intercropped maize (b, d) and soybean (a, c) during the growing seasons of 2013 and 2014. RI: one row maize intercropped with one row soybean; SI1: strip intercropping with maize model width (*W*_*m*_): soybean model width (*W*_*s*_) of 60:140 (maize row of 20 cm); SI2: strip intercropping with *W*_*m*_: *W*_*s*_ of 80:120 (maize row of 40 cm); SI3: strip intercropping with *W*_*m*_: *W*_*s*_ of 100:100 (maize row of 60 cm); SS: sole soybean; SM: sole maize. Bars at each data point represent standard deviation of the mean (n = 3).

There was no significant difference between maize plant height among treatments (Fig 5). The plant height of soybean in RI was significantly higher than that of monoculture. There was no significant difference of soybean height between SI1, SI2, and SI3.

### Intercepted PAR

**Fig 6:**
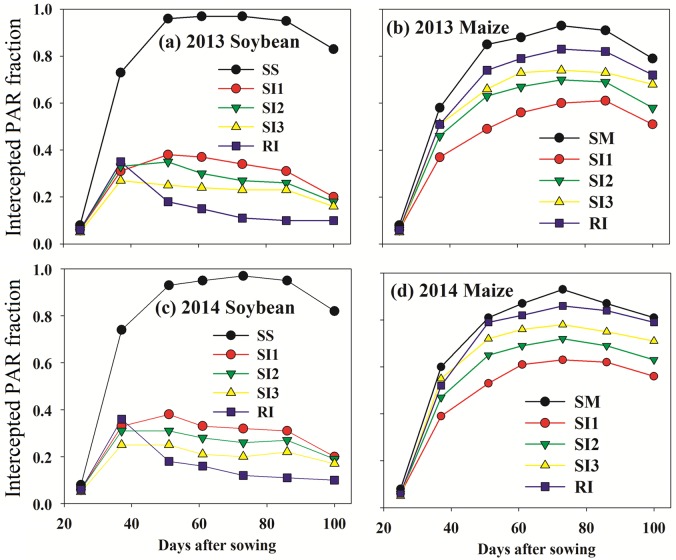
PAR intercepted fraction dynamics of intercropped maize (b, d) and soybean (a, c) during the growing seasons of 2013 and 2014. RI: one row maize intercropped with one row soybean; SI1: strip intercropping with maize model width (*W*_*m*_): soybean model width (*W*_*s*_) of 60:140 (maize row of 20 cm); SI2: strip intercropping with *W*_*m*_: *W*_*s*_ of 80:120 (maize row of 40 cm); SI3: strip intercropping with *W*_*m*_: *W*_*s*_ of 100:100 (maize row of 60 cm); SS: sole soybean; SM: sole maize. Bars at each data point represent standard deviation of the mean (n = 3).

The PAR intercepted fraction was related to the *LAI*, crop height, and intercropping configurations. The PAR intercepted fraction for intercropped soybean reached peak at 40–50 DAS, and 70 DAS for intercropped maize (Fig 6a and 6c). The average PAR intercepted fraction of soybean showed that SS (0.71)>SI1 (0.28)>SI2 (0.23)>SI3 (0.21)>RI (0.16) in 2013 and SS (0.71)>SI1 (0.26)>SI2 (0.23)>SI3 (0.19)>RI (0.16) in 2014. The average PAR intercepted fraction of maize showed that (Fig 6b and 6d) SM (0.65)>RI (0.61)>SI3 (0.56)>SI2 (0.50)>SI1 (0.44) in 2013 and SM (0.69)>RI (0.64)>SI3 (0.59)>SI2 (0.54)>SI1 (0.47) in 2014.

**Fig 7:**
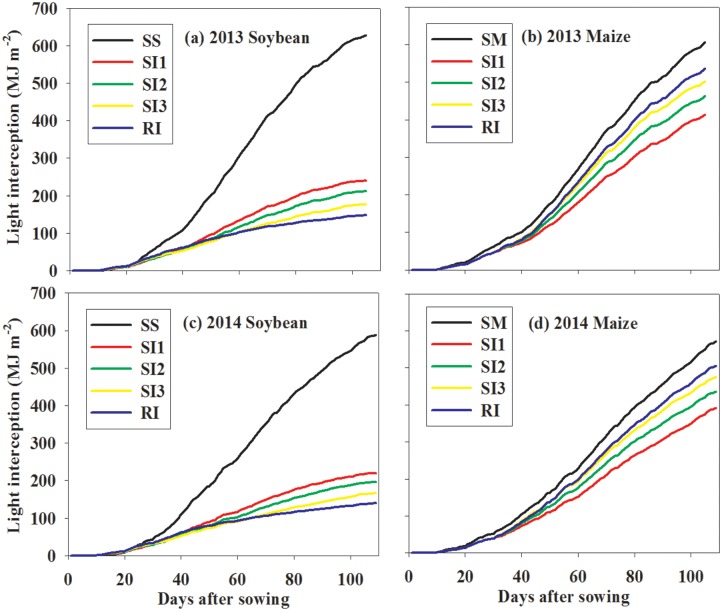
Accumulative intercepted PAR dynamics of intercropped soybean (a, c) and maize (b, d) during the growing seasons of 2013 and 2014. RI: one row maize intercropped with one row soybean; SI1: strip intercropping with maize model width (*W*_*m*_): soybean model width (*W*_*s*_) of 60:140 (maize row of 20 cm); SI2: strip intercropping with *W*_*m*_: *W*_*s*_ of 80:120 (maize row of 40 cm); SI3: strip intercropping with *W*_*m*_: *W*_*s*_ of 100:100 (maize row of 60 cm); SS: sole soybean; SM: sole maize.

For soybean (Fig 7a and 7c), the total intercepted PAR from sowing to maturity showed SS (509 MJ m^-2^)>SI1 (208 MJ m^-2^)>SI2 (173 MJ m^-2^)>SI3 (144 MJ m^-2^)>RI (128 MJ m^-2^). For maize (Fig 7b and 7d), it showed SM (598 MJ m^-2^)>RI (506 MJ m^-2^)>SI3 (475 MJ m^-2^)>SI2 (450 MJ m^-2^)>SI1 (403 MJ m^-2^).

### Radiation Use Efficiency (RUE)

The RUE of soybean showed that SI2>RI>SI3>SI1>SS (Table 1). And the RUE of maize was SI1>SI2>SI3>RI>SM, indicating that the RUE of intercrops were significantly higher than that of sole crops. The system RUE showed that SI2>SM>SI3>RI>SI1>SS because maize RUE were twice of soybean RUE.

**Table 1:** Dry matter, intercepted PAR and radiation use efficiency (RUE) of soybean, maize and system. The “System” combined all crops together. System intercepted PAR and dry matter is sum of that all crops, and system RUE is the system dry matter divided by system intercepted PAR. Means in columns followed by the different letters are significantly different (P<0.05; n = 3).

Year	Treatment	Soybean			Maize			System		
		Dry matter	Intercepted PAR	RUE	Dry matter	Intercepted PAR	RUE	Dry matter	Intercepted PAR	RUE
		g m^-2^	MJ m^-2^	g MJ^-1^	g m^-2^	MJ m^-2^	g MJ^-1^	g m^-2^	MJ m^-2^	g MJ^-1^
2013	SI1	420b	222b	1.89c	1848c	414d	4.46ab	2269c	636bc	3.57c
	SI2	398b	187c	2.13ab	2136b	463c	4.61a	2534a	650ab	3.90a
	SI3	312c	150d	2.08b	2185b	502b	4.35b	2497ab	652ab	3.83ab
	RI	294c	134d	2.19a	2204b	536b	4.11c	2498ab	670a	3.72b
	SS	742a	545a	1.36d				742d	545d	1.36d
	SM				2437a	617a	3.95d	2437b	617c	3.95a
2014	SI1	448b	194b	2.31b	2003c	392d	4.91a	2451b	586bc	4.19a
	SI2	413c	159c	2.59a	2093bc	437c	4.79b	2506ab	596b	4.20a
	SI3	308d	138d	2.23b	2196ab	482b	4.56c	2504ab	620a	4.04bc
	RI	291d	122d	2.28b	2284a	506b	4.51c	2574a	628a	4.10ab
	SS	756a	474a	1.59d				756d	474d	1.59d
	SM				2292a	580a	3.95d	2292c	580c	3.95c
Average	SI1	434b	208b	2.10b	1926d	403d	4.69a	2360b	611c	3.88b
	SI2	405b	173c	2.36a	2115c	450c	4.70a	2520a	623b	4.05a
	SI3	310c	144d	2.16b	2191bc	475bc	4.46b	2501a	619bc	3.94ab
	RI	292c	128d	2.29a	2244b	506b	4.31c	2536a	634a	3.91b
	SS	749a	509a	1.48c				749c	509e	1.48c
	SM				2364a	598a	3.95d	2364b	598d	3.95ab

The intercepted PAR of soybean showed SS>SI1>SI2>SI3>RI. The intercepted PAR of maize showed SM>RI>SI3>SI2>SI1, indicating the sole crop intercepted more PAR than intercrops. The system intercepted PAR showed that RI>SI2>SI3>SI1>SM>SS.

The dry matter of soybean showed the trend of SS>SI1>SI2>SI3>RI (Table 1). The dry matter of maize showed an increasing trend of SM>RI>SI3>SI2>SI1, and in SI1 it was significantly less than in other treatments. The dry matter of the intercropping systems was significantly higher than that of the monocultured systems (RI>SI2>SI3>SM>SI1>SS).

### Dry matter simulation and validation

**Fig 8:**
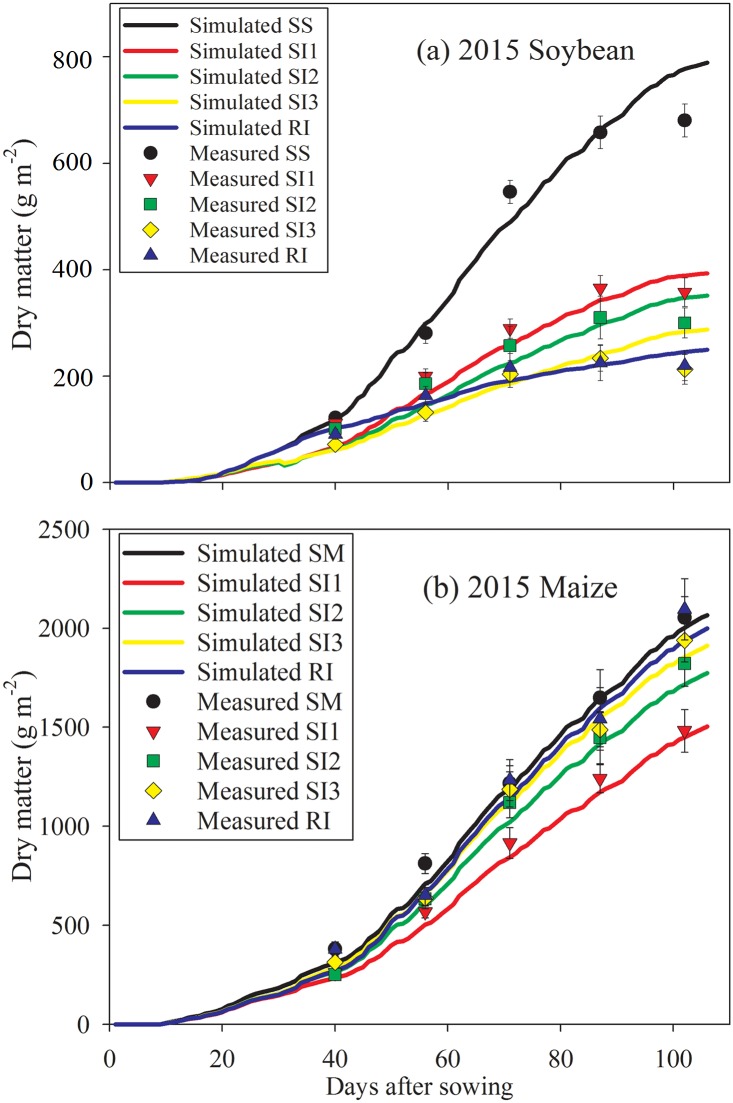
Dry matter simulation of intercropped soybean (a) and maize (b) during the growing seasons of 2015. The measured data were represented in dots. RI: one row maize intercropped with one row soybean; SI1: strip intercropping with maize model width (*W*_*m*_): soybean model width (*W*_*s*_) of 60:140 (maize row of 20 cm); SI2: strip intercropping with *W*_*m*_: *W*_*s*_ of 80:120 (maize row of 40 cm); SI3: strip intercropping with *W*_*m*_: *W*_*s*_ of 100:100 (maize row of 60 cm); SS: sole soybean; SM: sole maize. Bars at each data point represent standard deviation of the mean (n = 3).

In soybean, SS always caused higher dry matter level than the other treatments (Fig 8a). The RI resulted in higher dry matter level than the other intercropping treatments before 50 DAS. After 70 DAS, the dry matter showed a trend of SS>SI1>SI2>SI3>RI. In maize, it showed SM>RI>SI3>SI2>SI1 (Fig 8b). The simulation value basically reflected the true value. The RMSE and MBE of soybean were no more than 50 and 36 g m^-2^, respectively (Table 2). The RMSE and MBE of maize were no more than 70 and 59 g m^-2^, respectively.

**Table 2:** RMSE and MBE of simulated and measured dry matter of each treatment in 2015.

Treatment	Soybean		Maize	
	RMSE	MBE	RMSE	MBE
SS	49.02	35.92		
SM			60.99	50.25
SI1	32.71	31.91	56.66	53.13
SI2	36.13	34.23	66.61	53.83
SI3	33.12	21.95	59.95	55.94
RI	18.05	15.99	69.27	58.25

## Discussion and Conclusion

The HHLA model was widely used in intercropping systems with horizontally homogeneous leaf area [14–15], which had no obvious strips. The row intercropping (RI) treatment in our research is fit to use the HHLA model. However, HHLA model showed marked errors in analyzing the strip intercropping system [6]. The RCRT model [10, 23] and ERCRT [6] model were used to calculate the intercepted PAR of strip intercropping. In our study, ERCRT model was applied to calculate the intercepted PAR of strip intercropping configurations with different row arrangements.

The ERCRT model and HHLA model were used to evaluated the intercepted PAR of strip intercropping (2:2 maize-to-soybean rows, SI) and row intercropping with horizontally homogeneous leaf area (1:1 maize-to-soybean rows, RI), respectively. The architecture of the canopy, which was affected by crop densities, crop height, and row arrangement [12], was the deciding factor for crop intercepted PAR [11]. The separation of the maize upper canopy in strip intercropping led to more intercepted PAR of soybean compared to RI. Moreover, the great distance between maize strip was advantageous to the increase of intercepted PAR for short statured crop. Previous studies showed the same results [6, 11]. It is confirmed that the yield of short statured crop would be higher because of the greater distance between maize strips [7].

The radiation use efficiency (RUE) is another important factor for dry matter accumulation in addition to intercepted PAR. The RUE of short statured soybean in intercropping system was higher than that of sole cropping mainly due to the increase of diffused light and less light saturation in intercropping, which is similar to previous studies [11, 13, 16, 17]. Differed from previous studies, the RUE of tall statured maize of intercropping was higher than that of sole cropping in this research, which was due to the same density as sole maize cropping and the border row effect for both rows of intercropped maize plants. Above all, the RUE of intercropped maize and soybean in our research were higher than those reported by previous studies [13, 15]. The measured RUE was used in our study. The actual RUE of intercrops was related to various factors including the crop genotype, leaf photosynthetic capacity, field management, plant diseases, insect pests, rainfall, soil water content, and nutrition, *etc*. [6, 13, 16]. The models for intercepted PAR have been fully studied. However, further models are still needed to simulate RUE for different genotypes and ecotypes.

The crop intercepted PAR and RUE differed in different intercropping configurations. The RI had the highest system intercepted PAR due to the understory soybean reduced the light loss. However, the intercepted PAR of soybean in RI was far less than the other intercropping configurations, resulting in the reduction of soybean yield. In SI1 (strip intercropping with *W*_*m*_: *W*_*s*_ of 60:140), the narrower maize row had a negative effect on intercepted PAR of maize, and there might be light loss in the larger distance between maize row and soybean row. In SI3 (strip intercropping with *W*_*m*_: *W*_*s*_ of 100:100), the larger maize row limited the soybean intercepted PAR, and there might be light loss in maize rows. The system intercepted PAR of SI2 (strip intercropping with *W*_*m*_: *W*_*s*_ of 80:120) was higher than the other strip intercropping. Moreover, the RUE of both maize and soybean in SI2 were the highest in all configurations. Considering intercepted PAR and RUE, SI2 was the recommended configuration.

The research on models of intercepted PAR and RUE also provided an alternative method for estimation of dry matter. The low RMSE of simulated and measured value in our study illustrated the feasibility of our models. This method will provide a reference value of dry matter to improve the credibility of the experiment results. It also can be used in virtual experiment considering different row configurations of intercropping.

## Supporting Information

S1 Fig Fim_black_ and Fib_black_ in model construction. (TIF)

S2 Fig The Fim and Fib in model construction. (TIF)

S3 Fig PAR intercepted fraction for maize (F1–5 and F9). (TIF)

S4 Fig PAR intercepted fraction for maize (F6–8). (TIF)

S1 Table The calculation for Extinction coefficient of soybean and maize. (XLSX)
