# Supplementary figures and images for: PAR Interception and Utilization in Different Maize and Soybean Intercropping Patterns
Source: PLoS One. 2017 Jan 5;12(1):e0169218. doi: 10.1371/journal.pone.0169218 (PMC5215860; doi:10.1371/journal.pone.0169218)

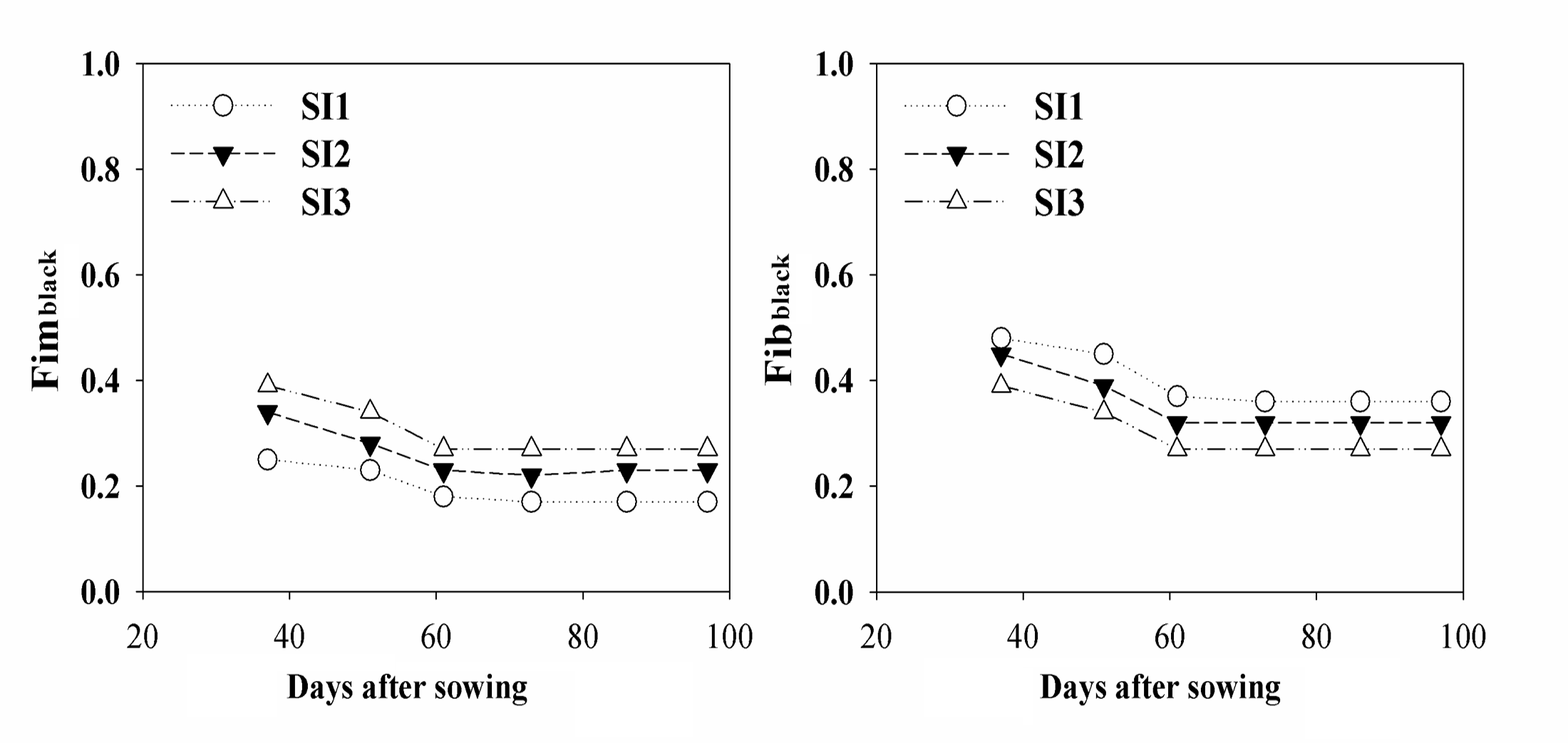

Supplement: S1 Fig — (TIF) [file pone.0169218.s001.TIF]

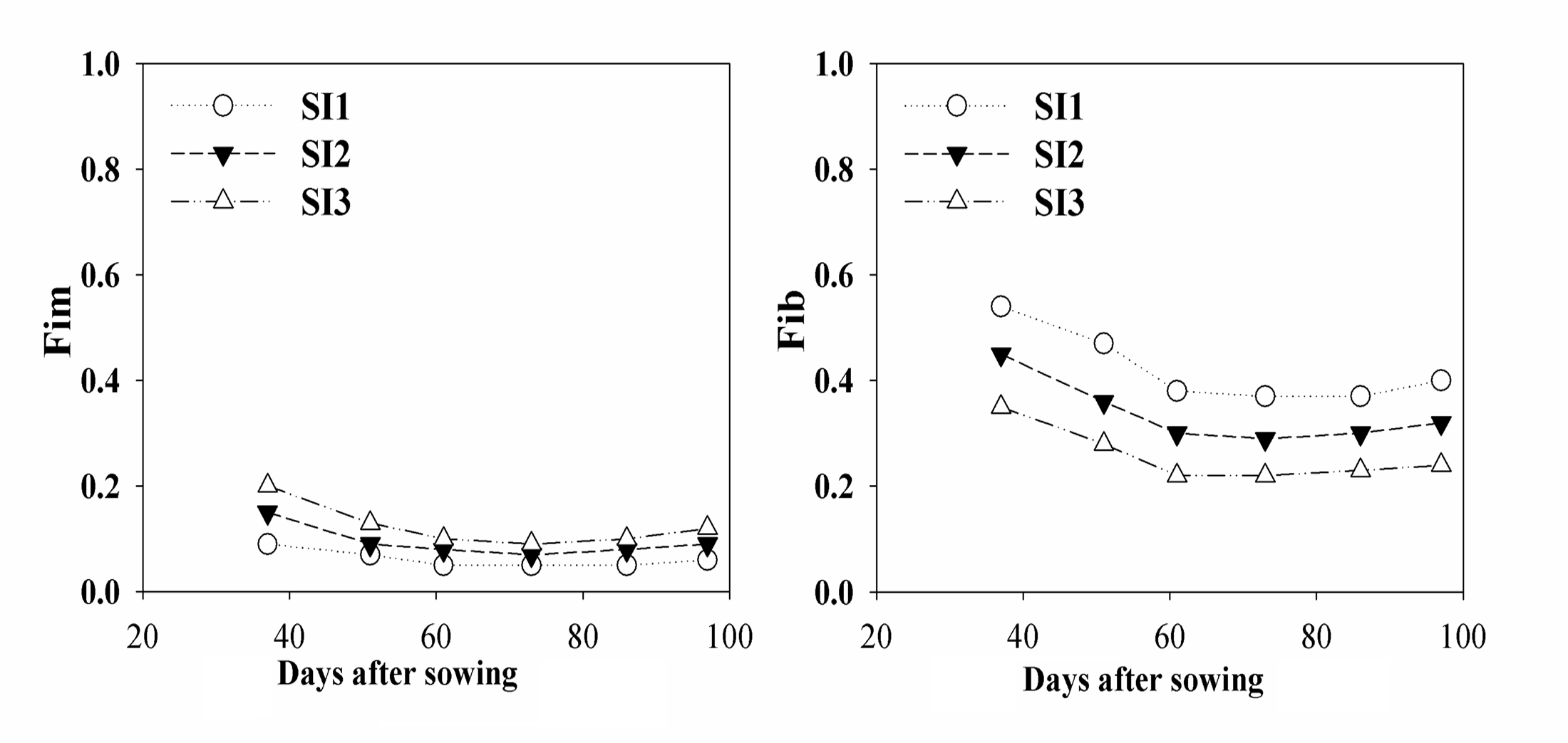

Supplement: S2 Fig — (TIF) [file pone.0169218.s002.TIF]

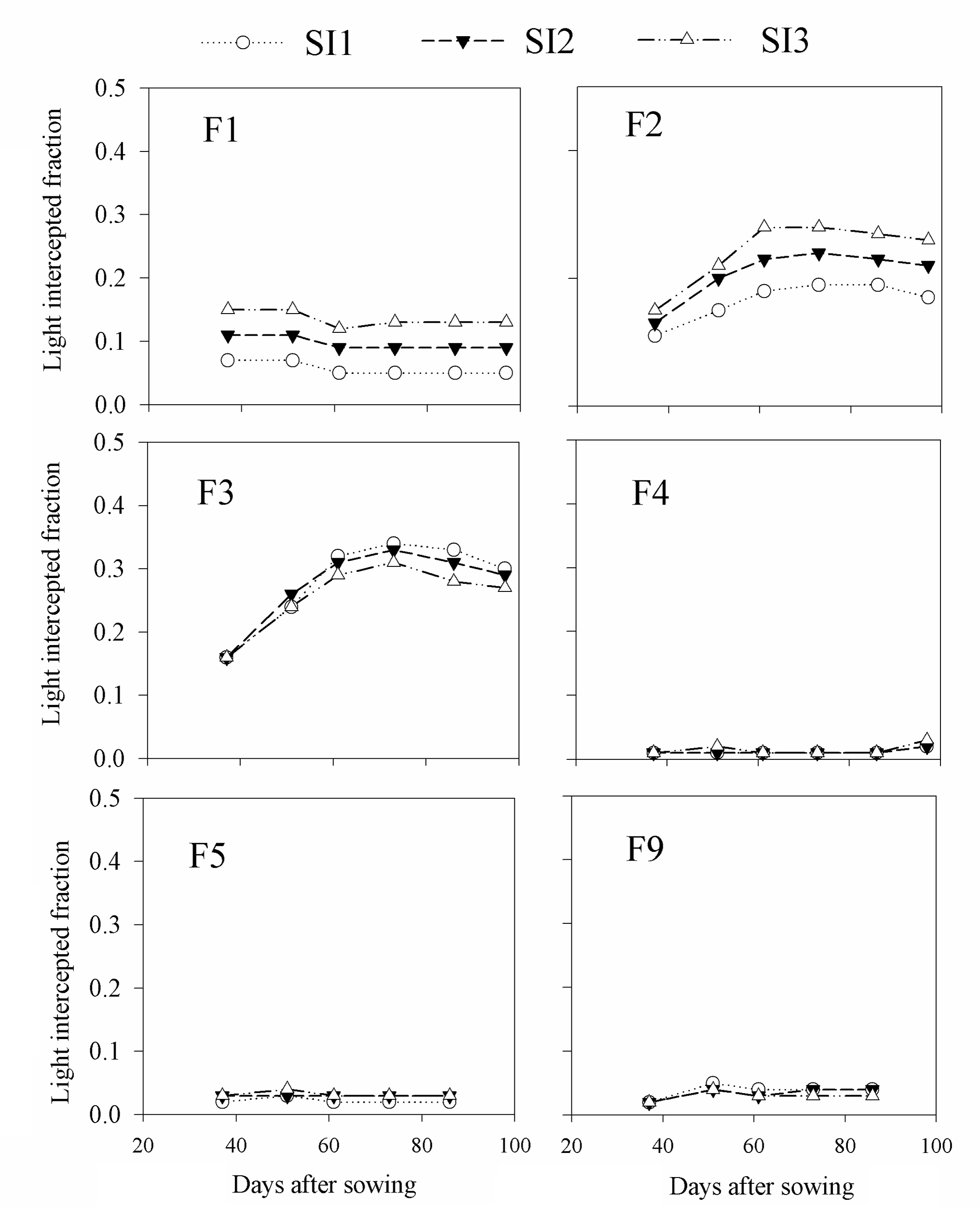

Supplement: S3 Fig — (TIF) [file pone.0169218.s003.TIF]

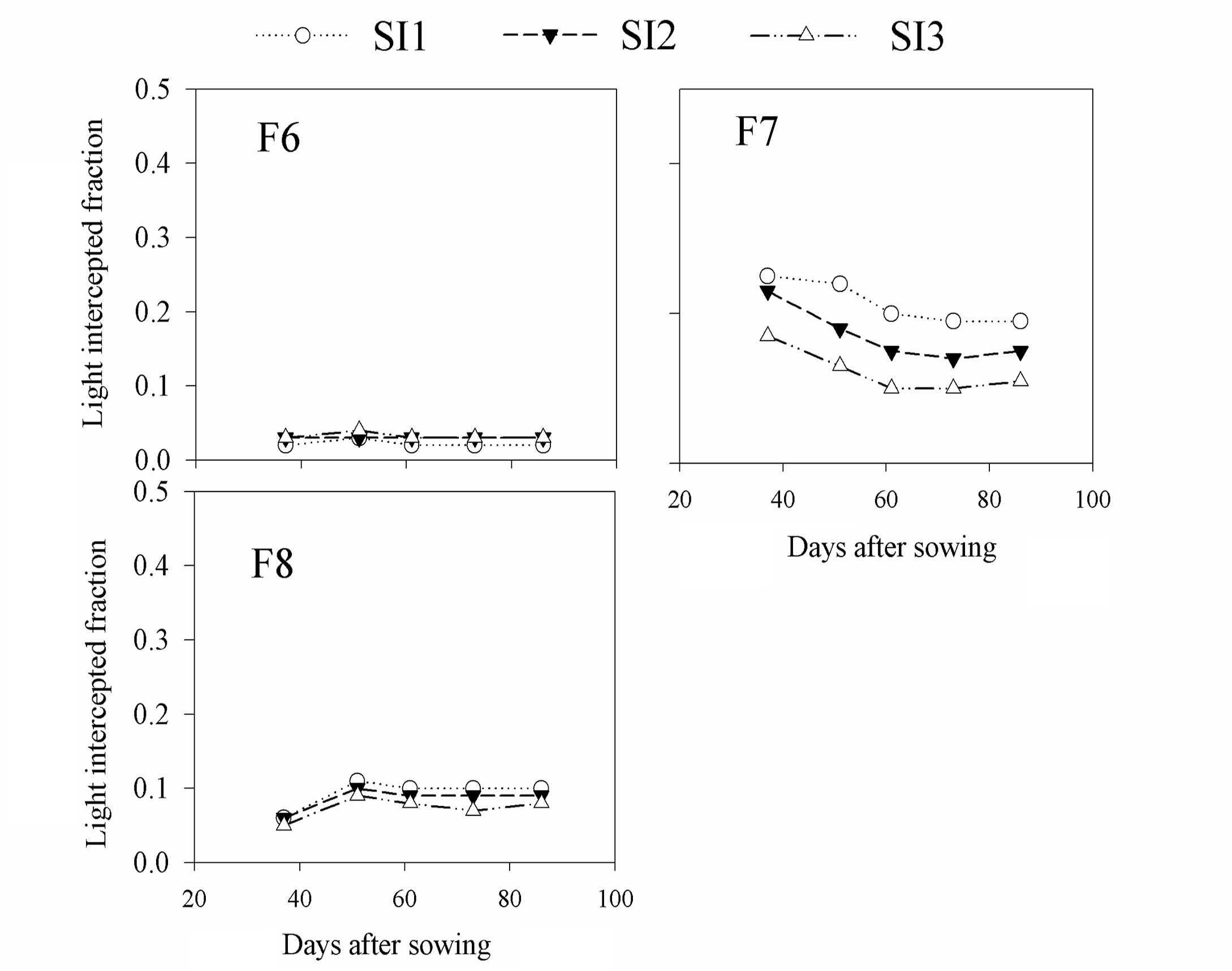

Supplement: S4 Fig — (TIF) [file pone.0169218.s004.TIF]
